# Effect of myo-inositol on the prevention of gestational diabetes in high-risk pregnant women: An RCT

**DOI:** 10.18502/ijrm.v23i4.18784

**Published:** 2025-06-10

**Authors:** Ashraf Moini, Mahboobeh Shirazi, Mahdi Sepidarkish, Maryam Rabiei, Afsaneh Tehranian, Arshia Shizarpour, Reihaneh Pirjani

**Affiliations:** ^1^Obstetrics and Gynecology Department, Arash Women's Hospital, Tehran University of Medical Sciences, Tehran, Iran.; ^2^Breast Disease Research Center (BDRC), Tehran University of Medical Sciences, Tehran, Iran.; ^3^Reproductive Epidemiology Research Center, Royan Institute for Reproductive Biomedicine, Tehran, Iran.; ^4^Obstetrics and Gynecology Department, Yas Complex Hospital, Tehran University of Medical Sciences, Tehran, Iran.; ^5^Cellular and Molecular Biology Research Center, Health Research Institute, Babol University of Medical Sciences, Babol, Iran.; ^6^Iranian Society of Perinatology, Tehran, Iran.; ^7^Obstetrics and Gynecology Department, Research Development Center, Arash Women's Hospital, Tehran University of Medical Sciences, Tehran, Iran.; ^8^Students' Scientific Research Center, Tehran University of Medical Sciences, Tehran, Iran.

**Keywords:** Gestational diabetes mellitus, Pregnancy, Myo-inositol, Primary prevention.

## Abstract

**Background:**

Gestational diabetes mellitus (GDM) is a rising problem which, if not diagnosed and treated in time, can lead to maternal, fetal, and neonatal complications. Therefore, it is very important to consider predisposing factors and prevention of GDM.

**Objective:**

This study aims to investigate the effect of myo-inositol (MI) on the prevention of GDM and other pregnancy outcomes.

**Materials and Methods:**

This randomized controlled trial was conducted at the Arash Women's hospital and Yas hospital Complex, Tehran, Iran between November 2019 and May 2020 and included 150 women; the study was divided into 2 groups (n = 75/each). Women received 4000 mg of MI plus 400 mg of folic acid daily in the MI group and 400 mg of folic acid in the placebo group from 11–14 gestational weeks for 14 wk. Participants underwent 75 gr oral glucose tolerance test at 24–28 wk and were followed up until delivery.

**Results:**

After adjustment for confounding factors, MI treatment was associated with a significant reduction of GDM (aRR: 0.58, 0.36, 0.91, p = 0.020). However, no significant difference was observed between the 2 groups in cases of other pregnancy outcomes and glycemia at each step of oral glucose tolerance test values.

**Conclusion:**

Our results showed that MI significantly reduced GDM. With insufficient evidences, more studies with an appropriate sample size are recommended.

## 1. Introduction

Gestational diabetes mellitus (GDM) is a rising problem which, if not diagnosed and treated in time, can lead to maternal, fetal, and neonatal complications, and even children born to diabetic mothers will be more resistant to insulin in childhood (1). Therefore, it is very important to consider predisposing factors and prevention of GDM and its timely diagnosis and treatment. Although lifestyle changes, especially diet and exercise, have always been considered as preventative factors for diabetes, many studies have been conducted to examine other contributing factors to GDM and its prevention so far.

Inositol, a natural sugar, and its isomers, including myo-inositol (MI) and d-chiro-inositol (DCI), have a potential effect in improving insulin sensitivity (2–4). The United States Food and Drug Administration introduced MI as a “generally recognized as safe” compound, and it has also been demonstrated to be safe in pregnancy (5). Although some previous studies have found MI to be effective in preventing GDM, given the existence of low numbers of clinical trials in this field, it has not yet been incorporated in obstetrics guidelines as a recommended method of preventing GDM. In contrast, the prevalence of diabetes and the influencing factors are different in various populations and ethnicities, hence, it seems that race and even food habits and culture may have an important role. Accordingly, it is valuable that drugs and supplements that may have a preventive effect on GDM be studied in different populations. Therefore, in this study, we aimed to investigate the effect of MI on the prevention of GDM in an Iranian population.

## 2. Materials and Methods

This randomized clinical trial was conducted between November 2019 and May 2020, on 138 high-risk pregnant women from 2 hospitals affiliated with Tehran University of Medical Sciences, Tehran, Iran (Arash Women's hospital and Yas hospital Complex).

Inclusion criteria were pregnant women in the first trimester (between 11 and 14 wk of gestation), body mass index (BMI) > 30 Kg/m^2^, family history of type 2 diabetes, previous history of GDM, polycystic ovary syndrome (PCOS), and history of infant macrosomia. Exclusion criteria included a history of diabetes mellitus, multiple pregnancies, acute infection during pregnancy, and impaired oral glucose tolerance test (OGTT) in first-trimester routine tests. Pregnant women who fulfilled the eligibility criteria were enrolled in this trial, prospectively stratified considering the recruitment center, and randomly assigned to receive either MI or placebo.

The stratified block randomization method was designed by a researcher who was unaware of the clinical condition of the participants using Stata software version 13. The number of blocks considered was 6, with an allocation of a 1:1 ratio.

Women in the MI group received 2000 mg MI plus 200 µg folic acid (Inofolic; Lo.Li. Pharma International, Rome, Italy) twice a day until 24–28 gestational weeks for a total of 14 wk, and women in the placebo group received 400 mg folic acid daily. All participants in both groups were given the same consultation in terms of exercise, nutrition, and lifestyle at the time of enrollment. In each visit, participants were followed up until delivery about potential side effects of the drug, such as headache, gastrointestinal problems, nausea, vomiting, insomnia, and tiredness, and information were collected by a data gatherer who was blinded to the recruitment process. In our study, the primary outcome was GDM. All women underwent 75-gr OGTT at 24–28 wk, and the test was interpreted based on the International Association of Diabetes and Pregnancy Study groups recommendation (6). GDM was diagnosed if one or more of the 3 values exceeded or equaled the threshold. The threshold values were 92 mg/dl, 180 mg/dl, and 153 mg/dl for fasting glucose, the 1 ${}^{\text{st}}$ and 2 ${}^{\text{nd}}$ hr after glucose load, respectively.

Secondary outcomes were macrosomia, pre-eclampsia, preterm labor (PTL), preterm premature rupture of membrane (PPROM), intrauterine growth restriction (IUGR), and delivery mode. An expert gynecologist assessed the fetuses for IUGR (ultrasound-estimated fetal weight is below the 10 ${}^{\text{th}}$ percentile for gestational age), pre-eclampsia (systolic blood pressure ≥ 140 mmHg or diastolic blood pressure ≥ 90 mmHg on 2 occasions at least 4 hr apart and proteinuria after 20 wk of gestation), PTL (birth before 37 wk of pregnancy), and PPROM.

Important changes to methods after trial commencement: we anticipated an 18-months for recruiting of the calculated women, but due to the COVID-19 pandemic, decreased number of prenatal routine visits, and also pregnant women's unwillingness to participate in the study due to the pandemic crisis, we were forced to reduce the sample size. Therefore, we decided to cease the recruitment process and analyzed the existing data. Given the final study size, we obtained 48% post power based on the primary outcome (GDM).

The simple randomization had a 1:1 ratio and was stratified per participating center. Participants, clinicians, investigators, and outcome assessors were blinded to the intervention allocation. We used sealed envelopes to conceal the allocation.

### Sample size

The sample size was calculated based on the primary outcome of the trial, the incidence of GDM. Based on the study of D'Anna et al. which reported a GDM incidence of 14.0% for MI group and 33.6% for control group (7), we used the following formula to calculate the sample size: 


$$N_{1}=\left\{z_{1}-\alpha /2*\sqrt{\bar{p}*\bar{q}*\left(1+\frac{1}{k}\right)}\right.$$

$${\left.+z_{1-\beta }*\sqrt{p_{1}*q_{1}*\left(1+\frac{1}{k}\right)}\right\}}^{2}/\Delta^{2}$$

$$q_{1}=1-p_{1}q_{2}=1-p_{2}\bar{p}=\frac{p_{1}+kp_{2}}{1+K}\bar{q}=1-\bar{p}$$

Considering a 5% alpha and 95% power to detect a 19.6% difference in primary outcome between 2 treatment groups, as well as 15% lost during follow-up, a sample size of 138 women per arm was calculated. Initially, the study was designed to enroll 278 participants, as registered on IRCT.ir. However, due to the unforeseen impact of the COVID-19 pandemic, recruitment was significantly troubled, and the study was completed with 145 participants. Despite this reduction in sample size, a post hoc power analysis was conducted to assess the study's statistical power with the achieved sample size. The analysis revealed that the study maintained sufficient statistical power (83%, based on the achieved adjusted odds ratio of 0.58 and a GDM incidence of 33.19% in the control group and alpha level of 0.05 using G*Power software) to detect the primary outcome. This suggests that the findings remain robust and interpretable within the context of the reduced sample size.

### Ethical Considerations

This trial was approved by the Ethics Committee of Tehran University of Medical Sciences, Tehran, Iran (Code: IR.TUMS.VCR.REC.1398.618) and registered in Iranian Registry of Clinical Trials (ID: IRCT20120826010664N4, May 8 ${}^{\text{th}}$ 2019). Changes were applied before the trial started. This study was reassessed by the mentioned ethics committee, and an update was made to the trial's IRCT profile on 2024–08-17. This trial was conducted in adherence to CONSORT guidelines. Written consent was obtained from all participants.

### Statistical Analysis

We used Stata 13 (Stata Corp, College Station, TX, USA) for statistical analysis, according to the intention-to-treat approach. We imputed missing data using multiple imputations using the chained equations method. Baseline and clinical characteristics were summarized using means ( ± standard deviation) and absolute frequencies (percentages). Continuous variables were compared between the intervention groups using an independent samples *t* test, while categorical variables were assessed with a Chi-square test. Before conducting statistical comparisons, normality was evaluated visually using quantile-quantile plots and further verified with the Kolmogorov-Smirnov test. For continuous outcomes measured over time, linear mixed-effects models were employed to examine within-group changes from baseline and between-group differences in those changes. The effect size was reported as an adjusted mean difference (aMD) with a 95% confidence interval (CI). For categorical pregnancy outcomes, a log-binomial regression model with robust standard errors was used to estimate risk differences between groups, expressed as an adjusted risk ratio (RR) with a 95% CI. The models adjusted for several covariates, including treatment group, gestational age, BMI, maternal age, prior cesarean delivery, history of pregnancy complications, pre-existing medical conditions, and delivery method. Statistical significance was set at p < 0.05.

## 3. Results

**Figure 1:**
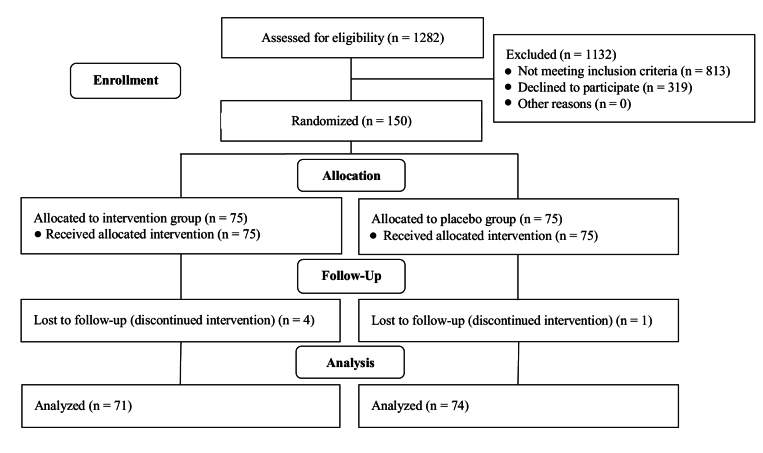
Participants flow diagram.

A total number of 1282 women were evaluated, of which 813 were not eligible according to the eligibility criteria. 319 women were not willing to participate in the study, mostly due to the COVID-19 crisis. Finally, 150 women (age range, 18–47 yr) were randomized into the study groups, 75 in each treatment group. All women except 4 in the MI group and one in the placebo group completed the study, and no one showed any adverse events due to the treatment received (Figure 1).

The 2 groups were comparable for baseline characteristics except for maternal age (mean difference [MD]: 2.01, 0.06, 3.94, p = 0.043) including pre-pregnancy BMI, nulliparous women, pre-existing hypertension, PCOS, macrosomia, and fasting glucose OGTT at the first trimester of pregnancy. Table I summarizes the main characteristics and clinical features of the study population at the baseline.

The incidence of GDM in the MI group (18/71, 25.35%) was identical to the placebo group (29/74, 39.19%) (RR: 0.64, 0.39, 1.05, p = 0.081). After adjustment for confounding factors such as gestational age, BMI, maternal age, family history of diabetes, and previous pregnancy problems, the MI treatment was associated with a significant reduction of GDM (adjusted risk ratio [aRR]: 0.58, 0.36, 0.91, p = 0.020). However, no significant difference was observed in glycemia at each step of OGTT values, either at basal values (aMD: -0.27, -3.08, 2.54, p = 0.821), in the 1 ${}^{\text{st}}$ hr (aMD: -2.41, -11.71, 6.88, p = 0.611) and 2 ${}^{\text{nd}}$ hr after consuming 75 gr glucose (aMD: -3.27, -12.72, 6.17, p = 0.497) between groups (Table II). No significant difference was highlighted between the 2 groups in cases of cesarean section, PTL, PPROM, IUGR, pre-eclampsia, polyhydramnios, and oligohydramnios (Table III).

**Table 1:** Baseline characteristics of the sample according to the intervention groups

**Variables**	**Myo-inositol (n = 71)**	**Placebo (n = 74)**	**P-value**
Age (yr)*	32.81 ± 5.57 33 (25, 36)	30.81 ± 6.22 30 (26, 36)	0.043
Pre-pregnancy BMI (kg/m^2^)*	27.76 ± 5.02 25.4 (23.9, 30.1)	27.06 ± 5.12 24.8 (21.6, 25.8)	0.409
Pre-pregnancy weight*	72.96 ± 13.73 73 (64, 80)	71.31 ± 14.34 71 (61, 79.25)	0.480
Gravidity*	2.04 ± 1.04 2 (1, 3)	2.04 ± 1.19 2 (1, 3)	0.993
Parity*	0.73 ± 0.86 1 (0, 1)	0.58 ± 0.74 0 (0, 1)	0.258
Live birth*	0.70 ± 0.83 1 (0, 1)	0.54 ± 0.68 0 (0, 1)	0.198
Miscarriage*	0.30 ± 0.62 0 (0, 0)	0.45 ± 0.81 0 (0, 0)	0.217
Gestational age at delivery (days)*	12.34 ± 1.08 12 (11.4, 13.2)	12.66 ± 1.05 12 (11, 13)	0.076
Waist circumference (cm)*	97.41 ± 10.41 98 (92, 104)	95.60 ± 10.41 95 (88, 103)	0.458
Hip circumference (cm)*	107.46 ± 9.45 107 (103, 113)	107.62 ± 9.62 109 (101, 114)	0.940
Waist/Hip ratio*	0.90 ± 0.06 0.89 (0.86, 0.95)	0.88 ± 0.07 0.88 (0.85, 0.91)	0.940
Pre-existing hypertension**	3 (2.67)	8 (10.81)	0.209
Pre-existing infertility**	13 (18.31)	14 (18.91)	0.209
Pre-existing PTB**	4 (5.63)	6 (8.11)	0.746
Pre-existing GDM**	12 (16.91)	10 (13.51)	0.570
Pre-existing IUFD**	2 (2.81)	3 (4.05)	0.999
Family history of DM**	49 (69.01)	55 (74.32)	0.209
*Data presented as Mean ± SD, median (interquartile range), independent *t* test. **Data presented as n (%), Chi-squared test. BMI: Body mass index, PTB: Preterm birth, GDM: Gestational diabetes mellitus, IUFD: Intrauterine fetal death, DM: Diabetes mellitus			

**Table 2:** OGTT glucose values and the incidence of GDM according to the intervention groups

**Variables**		**Myo-inositol**	**Placebo**	**Diff^#^**	**95% CI**		**P-value**
					**Lower**	**Upper**	
**Fasting glucose OGTT (mg/dL)**							
	**Pre**	82.93 ± 6.63	82.53 ± 5.70	0.40	-1.62	2.43	0.696 ${}^{†}$
	**Post**	83.56 ± 7.94	82.86 ± 9.59	-0.27	-3.08	2.54	0.851 ${}^{§}$
	**Change**	0.63 ± 10.37	0.33 ± 9.98	0.29	-3.04	3.63	0.861 ${}^{†}$
**1-hr glucose OGTT (mg/dL)**							
	**Pre**	139.09 ± 22.49	137.50 ± 26.79	1.59	-6.54	9.73	0.699 ${}^{†}$
	**Post**	154.86 ± 28.64	150.72 ± 33.31	-2.41	-11.71	6.88	0.611 ** ${}^{§}$ **
	**Change**	15.76 ± 31.11	13.21 ± 34.71	2.54	-8.28	13.38	0.643 ${}^{†}$
**2-hr glucose OGTT (mg/dL)**							
	**Pre**	105.80 ± 19.23	102.77 ± 16.91	3.03	-2.90	8.97	0.315 ${}^{†}$
	**Post**	125.39 ± 29.51	120.36 ± 29.55	-3.27	-12.72	6.17	0.497 ${}^{§}$
	**Change**	19.59 ± 32.97	17.59 ± 31.51	1.99	-8.58	12.58	0.710 ${}^{†}$
**Weight**							
	**Pre**	72.96 ± 13.73	71.31 ± 14.34	1.65	-2.96	6.26	0.480 ${}^{†}$
	**Post**	79.59 ± 14.08	77.09 ± 13.80	-0.61	-2.09	0.87	0.419 ${}^{§}$
	**Change**	6.86 ± 4.71	6.60 ± 4.38	0.25	-1.28	1.79	0.742 ${}^{†}$
**Blood pressure systolic**							
	**Pre**	112.40 ± 10.26	110.26 ± 12.28	2.13	-1.58	5.85	0.259 ${}^{†}$
	**Post**	111.45 ± 11.19	107.97 ± 9.40	-2.52	-5.53	0.49	0.101 ${}^{§}$
	**Change**	-0.94 ± 13.05	-2.29 ± 11.53	1.35	-2.68	5.39	0.509 ${}^{†}$
**Blood pressure diastolic**							
	**Pre**	70.14 ± 8.97	68.27 ± 10.01	1.87	-1.25	4.99	0.239 ${}^{†}$
	**Post**	70.63 ± 9.31	68.36 ± 8.24	-1.61	-4.24	1.01	0.228 ${}^{§}$
	**Change**	0.49 ± 10.95	0.09 ± 10.80	0.39	-3.17	3.97	0.826 ${}^{†}$
Data presented as Mean ± SD. ${}^{†}$ *t* test, ${}^{§}$ linear mixed-effects model (the included variables were: Gestational age, body mass index, maternal age, previous cesarean section, previous pregnancy problems, pre-existing medical problems, and mode of delivery. ${}^{\#}$ Intervention minus control group. OGTT: Oral glucose tolerance test, GDM: Gestational diabetes mellitus, CI: Confidence interval							

**Table 3:** Outcomes in women randomized to receive myo-inositol treatment and placebo

**Pregnancy outcome**	**Crude RR (95% CI)**	**P-value**	**Adjusted RR (95% CI)†**	**P-value**
**GDM**	0.64 (0.39, 1.05)	0.081	0.58 (0.36, 0.91)	0.020
**CS**	0.96 (0.77, 1.19)	0.731	0.93 (0.76, 1.14)	0.521
**PTB**	1.63 (0.67, 3.98)	0.277	1.48 (0.61, 3.57)	0.376
**PROM**	1.30 (0.54, 3.11)	0.552	2.14 (0.84, 5.41)	0.108
**IUGR**	1.25 (0.39, 3.91)	0.701	1.24 (0.39, 3.94)	0.707
**Pre-eclampsia**	0.69 (0.11, 4.03)	0.685	0.55 (0.09, 3.26)	0.517
**Polyhydramnios**	0.78 (0.18, 3.36)	0.741	0.73 (0.16, 3.19)	0.680
**Oligohydramnios**	1.04 (0.15, 7.20)	0.967	1.09 (0.15, 7.66)	0.930
GDM: Gestational diabetes, CS: Cesarean section, PTB: Preterm birth, IUGR: Intrauterine growth restriction, PROM: Premature rupture of membranes, RR: Risk ratio, CI: Confidence interval. †Risk ratio estimated directly from Log binomial regression considering the type of outcome. The final multivariable models were adjusted for the following risk factors: Gestational age, body mass index, maternal age, previous cesarean section, previous pregnancy problems, pre-existing medical problems, and mode of delivery				

## 4. Discussion

In this randomized clinical trial, we found that in women with at least one risk factor for GDM who had taken 4 gr MI supplement daily, the incidence of GDM was significantly reduced after adjusting for confounding factors; however, other pregnancy outcomes were not affected by MI supplement.

MI is a cyclic carbohydrate with 6 hydroxyl groups made by kidneys and in much lower amounts by liver and brain, and also its dietary intake is 500–1000 mg per day. Some foods rich in MI include almonds, citrus fruits, walnuts, oats, and beans (2). MI is necessary for cellular development, surveillance and growth, and is also a component of cell membranes due to its role in the structure of phosphatidylinositol. Neurotransmitter, osmotic regulation, signal transduction mediator for several hormones including insulin, follicle-stimulating hormone, and thyroid-stimulating hormone, are some of the functions of MI in the human body (2, 3). Insulin uses inositol phosphoglycans as a secondary mediator for oxidative and non-oxidative glucose metabolism and also controls glucose absorption. MI and DCI also play a role in storing glucose as glycogen (2). MI inhibits glucose absorption by competing with the same transporter system in the duodenum and, thereby, helps reduce blood glucose levels (5, 8). Increased blood glucose levels and insulin resistance reduce the absorption and biosynthesis of MI in tissues. Glucose also increases its urinary excretion by inhibiting the renal reabsorption of MI. Changes in plasma and urinary levels of MI may be the first signs of insulin resistance (2). Considering the above, MI deficiency can lead to increased insulin resistance, impaired antioxidant defense, and increased oxidative stress.

Inositol imbalances can impair fertility and complicate pregnancy; therefore, inositol supplementation may prevent or treat pregnancy-related pathologies (3). According to an important role of MI in the human body, various studies have investigated its effects on conditions such as infertility, ovulation induction, in vitro fertilization, PCOS, regularization of menstrual cycle, diabetes, and GDM.

Since MI plays a key physiological role in transmitting intracellular insulin signals, a number of investigations have been conducted about the effect of MI on GDM during recent years. One of the first studies in this area was a study by D'Anna et al. which provided the first insight into the preventive effect of MI on GDM (7). However, studies in this area remain relatively limited. A systematic review and meta-analysis indicated that by 2024, approximately 7 clinical trials have been conducted to investigate the effects of myo-inositol/inositol on the prevention of GDM (9).

Despite the small number of clinical trials about the effect of MI on GDM, several systematic reviews and meta-analyses have been published in this field in recent years (10–14), and it is noteworthy that almost all of the studies included in them are common and their number is limited (7, 15–20). Interestingly, most of these studies were conducted in Italy (7, 15, 16, 19, 20).

Although inclusion criteria in different studies, the duration of MI administration, and even gestational age at starting medication have been varied among them; however, in most of them, MI has been effective in preventing GDM and a meta-analysis showed that prophylactic use of MI reduces the risk of GDM by 61% (9). As well, other meta-analysis concluded that inositol consumption was associated with a significant reduction in GDM and preterm delivery; however, no significant difference was observed in the rate of cesarean section and shoulder dystocia, and neonatal consequences such as birth weight, macrosomia, neonatal hypoglycemia, and neonatal intensive care unit admission (14).

In a study (19), taking MI from the first trimester until delivery led to a 67% risk reduction in the incidence of GDM in overweight pregnant women, and in another study with the same researchers, MI administration from the first trimester to delivery reduced the risk of GDM in obese pregnant women (7). Also, a study on women with a parent with diabetes showed that taking MI from the first trimester of pregnancy led to a significant reduction in GDM and macrosomia; however, other pregnancy outcomes were not affected (16). Also, in a study on overweight non-obese women, 2 gr MI twice a day from the first trimester of pregnancy until 3 wk after delivery caused a significant reduction in the incidence of GDM in comparison with placebo (21). On the other hand, in a study from Ireland, MI in combination with DCI has been examined for preventing GDM, and it was found that using inositol did not reduce GDM incidence in pregnant women who have a family history of diabetes (18).

In our study, although the incidence of GDM was significantly lower in the MI group than in the control group, none of the glucose values in OGTT steps were significantly different between the 2 groups (Table II). Similar results have been obtained in a number of other studies (19, 21); however, in one study, all OGTT glucose values were lower in the MI group (7), and in another study, although fasting glucose and 1 ${}^{\text{st}}$ hr glucose values were significantly lower, 2 ${}^{\text{nd}}$ hr glucose values were not significantly different between 2 groups (16).

The duration of MI use was different among studies, and in most of them, the duration of taking MI is longer than in our study. Given the decreased incidence of GDM in our study, as in previous studies, it seems that long-term use of MI is not necessary to prevent GDM in high-risk individuals. Of course, further studies are needed in this area.

It has been mentioned that in some people, oral inositol is weakly absorbed, so they express a poor response to MI supplements and are recognized as “inositol-resistant” (4, 5). These might be areas for differences in the results of various studies. Interestingly, most of the studies that found MI to be effective in preventing GDM were conducted in Italy (7, 15, 16, 19, 20), and a study in Ireland found that the drug was ineffective in preventing GDM (18). In a study in northern Iran on 60 overweight participants, it was observed that receiving MI from 14–24 wk of gestation significantly reduced the incidence of gestational diabetes, the results of which are similar to ours (17). Accordingly, genetics, race, culture, and even food habits in different populations may have a role in this regard, so it is recommended that further studies be conducted in different populations to clarify the role of MI in preventing GDM.

In terms of MI safety, a systematic review disclosed no adverse outcomes in the women and no congenital anomalies in the newborns (14). MI doses of 18 gr for 3 months or 4 gr for one year are safe and tolerable (2). A Cochrane review also stated that no adverse events were observed related to MI supplementation during pregnancy (22). Moreover, as opposed to metformin, which shows some gastrointestinal symptoms, MI is well tolerated in pregnancy (4, 23). If future studies provide sufficient evidence about the preventive and therapeutic effect of MI on GDM, widespread use of this drug in the general population will be possible and may lead to minimizing the use of other drugs such as insulin and also metformin, which is not approved by the United States Food and Drug Administration for using in pregnancy. A multicenter, placebo-controlled, pilot randomized trial was conducted at 5 UK National Health Service hospitals to investigate the feasibility and acceptability trial on MI in pregnancy and concluded that trial on MI to prevent GDM is feasible (24). So, we hope that more studies will be conducted in this area in the future.

### Strengths and limitations

One of the limitations of our study was the insufficient sample size, which was mostly due to the COVID-19 pandemic crisis and the consequent reluctance of pregnant women to participate in the study. Moreover, given that the appearance of the drugs was different in the 2 study groups (inofoic powder and folic acid tablets), our study was open-label which is a limitation. Moreover, the strength of our study is that it has been implemented as a double-centric study.

## 5. Conclusion

In our study, consuming MI supplements from 11–14 wk of gestation with a dose of 2 gr twice a day for 14 wk significantly reduced the incidence of GDM in women who had at least one risk factor for GDM. However, other pregnancy outcomes, including PTL, PPROM, IUGR, pre-eclampsia, polyhydramnios, oligohydramnios, and also glucose values in OGTT steps were not affected.

Given that the evidences are still insufficient to support MI supplement as a preventative factor for GDM, conversely, previous studies have shown the safety and tolerability of this supplement during pregnancy. It is recommended that more studies with appropriate sample size be designed and implemented to evaluate the effect of MI on prevention and even treatment of GDM in different racial populations.

##  Data Availability

Data supporting the findings of this study are available upon reasonable request from the corresponding author.

##  Author Contributions

A. Moini and R. Pirjani designed the study. A. Moini, M. Shirazi, R. Pirjani, M. Rabiei, A. Shizarpour, and A. Tehranian collected the data. M. Sepidarkish participated in the study by data analysis and interpretation. All authors approved the final version.

##  Conflict of Interest 

The authors declare that there is no conflict of interest.
